# Stem Cell-Derived Extracellular Vesicles in the Treatment of Cardiovascular Diseases

**DOI:** 10.3390/pharmaceutics16030381

**Published:** 2024-03-11

**Authors:** Jennifer McDonald, Sidhesh Mohak, Zsolt Fabian

**Affiliations:** 1 School of Medicine and Dentistry, Faculty of Clinical and Biomedical Sciences, University of Central Lancashire, Fylde Road, Preston PR1 2HE, UK; jennifermcdonald@students.aucmed.edu; 2 Department of Internal Medicine, South Texas Health System, McAllen, TX 78503, USA; smohak@mail.sjsm.org

**Keywords:** stem cell-derived extracellular vesicles, exosomes, microvesicles, cardiovascular diseases, regenerative medicine

## Abstract

Cardiovascular disease constitutes a noteworthy public health challenge characterized by a pronounced incidence, frequency, and mortality rate, particularly impacting specific demographic groups, and imposing a substantial burden on the healthcare infrastructure. Certain risk factors, such as age, gender, and smoking, contribute to the prevalence of fatal cardiovascular disease, highlighting the need for targeted interventions. Current challenges in clinical practice involve medication complexities, the lack of a systematic decision-making approach, and prevalent drug therapy problems. Stem cell-derived extracellular vesicles stand as versatile entities with a unique molecular fingerprint, holding significant therapeutic potential across a spectrum of applications, particularly in the realm of cardio-protection. Their lipid, protein, and nucleic acid compositions, coupled with their multifaceted functions, underscore their role as promising mediators in regenerative medicine and pave the way for further exploration of their intricate contributions to cellular physiology and pathology. Here, we overview our current understanding of the possible role of stem cell-derived extracellular vesicles in the clinical management of human cardiovascular pathologies.

## 1. Introduction

Cardiovascular diseases (CVDs) are the leading cause of mortality globally, being responsible for about 17.9 million deaths and representing 32% of all global mortality in 2019. The majority of these, approximately 85%, were due to heart attacks and strokes [1,2]. Recent studies providing in-depth insights into the mortality rates, risk factors, and regional trends of CVDs, drawing data from 204 countries and territories, show that ischemic heart disease continues to be the predominant cause of CVD mortality worldwide, with an age-standardized rate of 108.8 deaths per 100,000 people [3]. Notably, the global death toll from CVDs rose from 12.4 million in 1990 to 19.8 million in 2022, a trend attributed to population growth, aging, and preventable risk factors. However, this increase in CVD mortality is not uniform across all regions. A marked disparity exists in CVD mortality rates between different geographical areas. For instance, Eastern Europe has the highest age-standardized total CVD mortality rate at 553 deaths per 100,000, in stark contrast to Australasia, which has the lowest at 122.5 deaths per 100,000 [3].

A key factor in these regional disparities is the presence of modifiable risk factors that significantly impact the prevalence and severity of cardiovascular diseases. Some of the leading modifiable risk factors for CVD deaths in 2021 included raised blood pressure, responsible for 10.8 million deaths, air pollution (4.8 million deaths), elevated LDL cholesterol (3.8 million deaths), and tobacco use (3.0 million deaths) [1]. From 2017 to 2020, a total of 127.9 million manifestations of CVD have been reported among adults in the United States. In 2020, coronary heart disease (CHD), the predominant contributor to CVD mortality, accounted for 41.2% of deaths, followed by stroke as the second leading cause, which contributed to 17.3% of all CVD fatalities [4]. In 2021, a similar trend was seen among the 3.4 million US resident deaths, with heart disease in the top position and stroke ranking fifth in the hierarchy, highlighting the critical importance of CVD in public health [5].

In contemporary clinical cardiovascular medicine, several challenges persist, prompting exploration into novel therapeutic approaches that might address these gaps. Standard clinical interventions, like primary coronary angioplasty or drug therapy, still face challenges in reducing elevated mortality rates [6,7]. Indeed, drug therapy problems, characterized by unintended treatment effects, the necessity for additional drug therapy and noncompliance to conventional medications are prevalent among heart failure patients [8,9]. The heightened complexity, increased utilization, and rising costs of pharmaceuticals contribute to challenges in drug selection, dosing, and monitoring, underscoring the need for improved clinical applications. One of the promising avenues to address the existing challenges in the clinical practice of cardiovascular diseases is regenerative medicine. Indeed, extensive research has been undertaken over the last two decades towards the implementation of stem cell-based cellular therapy in the clinical management of pathologies affecting the cardiovascular system [10].

## 2. Cellular Therapy

Cellular therapy, a broad field of medicine, uses cells to prevent, treat, or reduce further progression of disease. Via manipulation and subsequent transplantation of cells to restore or improve the function of degenerative tissues or organs, cellular therapy harnesses the unique properties of different types of cells to achieve these therapeutic effects [11,12]. There are various types of cellular therapies under this umbrella, including immunotherapy, gene therapy, platelet-rich plasma therapy, and stem cell therapy, all of which are utilized in different facets of medicine.

### Stem Cell-Based Cellular Therapy

Stem cell-based cellular therapy harnesses the inherent self-renewal and differentiating capacities of stem cells, often through ex vivo culturing, enabling clinicians and researchers to guide cell lineage progression based on the specifics of tissues requiring repair [13,14]. Stem cells are versatile cells which possess the abilities to both self-renew over extended periods of time and to generate specialized somatic cells [15,16]. They fall under totipotent, pluripotent, and multipotent categories, determined by their capacity to form diverse tissues. Totipotent stem cells, present briefly during early embryogenesis, can give rise to any cell type, including placental and umbilical cord cells, but are limited to the initial mitotic cell divisions at the zygote stage [17]. Pluripotent embryonic stem cells emerge during the blastocyst stage, following zygote formation, and can differentiate into any cell type except placental and umbilical cord species. Multipotent stem cells, also called adult or somatic stem cells, specialize in tissue-specific cell lines, with some remaining dormant in the G_0_ cell cycle phase [18]. Mesenchymal stromal cells (MSCs), a distinct somatic stem cell population found in connective tissues, particularly in the bone marrow, exhibit significant plasticity, differentiating into certain tissues, like fat, bone, or cartilage [19]. The bone marrow, hosting stromal MSCs and hematopoietic stem cells (HSCs), is a notably rich stem cell niche utilized routinely for therapeutic purposes [20]. In the past two decades, a novel form of pluripotent cells, termed induced pluripotent stem cells (iPSCs), has also been invented. These are species generated from differentiated somatic cells via in vitro reprogramming at the molecular level to regenerate various cell lines beyond their normal boundaries, behaving similarly to embryonic stem cells [21]. Toti-, pluri-, and multipotent stem cells share common traits, including self-renewal capacity, broad differentiation abilities, and extensive extracellular vesicle production and transport to surrounding tissues, holding substantial medical potential.

Multipotent stem cells, in particular, are viewed as promising tools for addressing various diseases, such as ischemic stroke, hypertrophic cardiomyopathy, and cardiorenal syndrome [22,23,24]. These tissue-resident, somatic stem cells, which are inherently multipotent, are present ubiquitously in various tissues where they remain in the G_0_ cell cycle phase until replication is required, responding to tissue-specific needs, such as liver regeneration, nerve repair, or renal tissue recovery. The underlying principle of utilizing stem cell species for regenerative purposes involves the transplantation of multipotent stem cells to damaged tissues, where they facilitate repair mechanisms through direct or—in the case of EV-focused studies—indirect effects. This seems to be particularly promising in the cardiovascular context due to the limited cellular replication of cardiomyocytes when regeneration is required [25].

Stromal cells derived from bone marrow, adipose tissue, umbilical cord blood, or Wharton’s jelly are considered primary targets for therapeutic purposes due to their diverse lineage specialization. Bone marrow-derived mesenchymal stromal/stem cells (BM-MSCs) seem to be particularly advantageous in clinical investigations, offering easy and standardized extraction, the potential to differentiate into various specialized cells, and immunomodulatory properties for broader applications.

Despite extensive research, aside from HSCs for hematological disorders, stem cell therapy remains relatively limited in medical practice [26,27,28,29,30]. A significant challenge in stem cell-based cellular therapy revolves around the acquisition of stem cells in both sufficient quality and quantity for seamless ex vivo manipulations and complication-free transplantation [14,21]. Indeed, transplantation techniques using adult stem cells can be applied to either autologous or allogeneic stem cell species. Autologous stem cells, derived from the patient, offer the advantage of immunological compatibility with recipient tissues [31]. However, the yield of isolated cells is typically limited, necessitating prolonged in vitro expansion, which increases the risk of cellular senescence and tumorigenesis [32,33,34]. Conversely, allogeneic stem cells, collected from donors, provide off-shelf availability but may trigger immune responses due to their foreign origin. Moreover, there is growing evidence that allogeneic stem cells can induce immunogenic memory responses, potentially hampering subsequent transplantations [35,36].

Many of the challenges of using stem cells can be sidestepped by utilizing stem cell-secreted products as an alternative. Indeed, a number of beneficial effects of stem cells in tissue regeneration were reported to be indirect suggesting the existence of cell-free mediators [37,38]. One of the candidate mediators of these indirect effects are stem cell-secreted extracellular vesicles (SCEVs) [39].

## 3. Extracellular Vesicles

Prior to the mid-1960s, when electron microscopy was able to identify sub-micron-sized biological entities, today known as extracellular vesicles (EVs), the origins of EV research unknowingly began in 1945 with coagulation experiments that suggested the existence of “a variety of minute breakdown products” of blood cells, now recognized as the EV fraction [40,41,42,43]. Several years later in 1967, Wolf researched the supernatant fraction from plasma-centrifugation and described it as a material that, although it originated from them, still differed from intact platelets [44]. Images of these extracellular matrix-fated “microparticles” obtained from platelet-free plasma were published for the first time in 1971 by Crawford, where he also demonstrated the cargo-carrying capacity of these vesicles by listing lipids, ATP, and contractile proteins as a few components [45]. Although over the last few decades these microparticles have been investigated under various names, like exosomes, ectosomes, or oncosomes, they are currently generally termed extracellular vesicles according to the most recent working guidelines [46]. A generic protocol of EV purification is provided as Supplementary Material [47].

Extensive research on EVs revealed the existence of an intracellular sorting/trafficking pathway, which was later used to describe the release of exosomes following the fusion of multivesicular bodies (MVBs) with the apical plasma membrane [48,49]. This laid the foundation for EV research over the next few decades, some of which included key aspects of EVs, like lateral diffusion in vesicle membranes [50], flippase functions [51], and key components, such as Rab, ARF, and tetraspanins [52,53]. Identification of the transferrin receptor and nucleoside transporters on exosomes further expanded our understanding of EVs with the concept that the composition of EVs can vary under distinct stress conditions, like disrupted iron homeostasis [54]. These data revolutionized our understanding of EVs, revealing their significant roles in cell maturation and physiological processes, rather than just being cellular waste. Indeed, EVs are shed into the extracellular space either constantly or in response to external stimuli that trigger intracellular pathways/changes, culminating in EV release. In platelets, for instance, their activation via thrombin/collage/calcium ionophore leads to microparticle release into the extracellular space. Similar effects have been reported in response to hypoxia, oxidative stress, aging, inflammation, or apoptosis in a wide range of cells, including stem cell species [55,56,57].

The idea that EVs could have therapeutic and physiological roles was first raised in the 1990s by observations of increased microparticles in transient brain ischemia and other infarctions, angina, or Crohn’s disease [58,59,60]. The finding that immune cell EVs can present antigens led to investigations of the potential of EVs in anti-tumor vaccines and dendritic cell-secreted EVs loaded with tumor peptides for cancer treatment, underlying the potential use of EVs in clinical practice [61,62,63].

### 3.1. Classification of EVs

Although there exist several classes of them, exosomes and microvesicles (MVs) are the two main subtypes of EVs [64]. Exosomes range in size from 30 to 100 nm and are released when multivesicular bodies (MVBs) loaded with intraluminal vesicles (ILVs) merge with the plasma membrane. For this reason, exosomes can be considered “intracellular vesicles” while they reside inside MVBs, hence the interchangeability between the terms ILVs and exosomes [65,66].

The primary process for the formation of MVBs and ILVs involves the endosomal sorting complex required for transport (ESCRT). This complex is made up of around thirty proteins organized into four sub-complexes (ESCRT-0, -I, -II, and -III) and includes highly conserved associated proteins, like VPS4, VTA1, and ALIX/PDCD6IP [67,68,69]. ESCRT-0 is responsible for identifying and isolating ubiquitinated transmembrane proteins in the endosomal membrane [70]. ESCRT-I and -II complexes are believed to play a key role in bending the membrane into buds containing sorted cargo, while components of ESCRT-III are crucial for the final separation of the vesicle [71,72,73,74]. ESCRT-0 is composed of HRS, which detects monoubiquitinated cargo proteins and links with STAM (the other ESCRT-0 component), Eps15, and Clathrin [75]. However, ESCRT-independent methods can also lead to the formation of MVBs and their ILVs [65]. Indeed, MVB biogenesis and ILV formation including cargo loading have all been observed in the absence of functional ESCRT complexes as well [76,77]. The lipid metabolism enzymes nSMase and PLD-2 are the potential mediators of the ESCRT-independent exosome formation, facilitating the inward budding and, therefore, ILV formation without the need for ESCRT complexes [78,79].

Microvesicles (MVs), in contrast, are vesicular structures between 0.1 and 1.0 μm in size, formed by the outward bulging of the plasma membrane. This direct shedding from the plasma membrane allows them to carry membrane markers from their parent cell [65,66,80]. Formation of MVs is influenced by a combination of factors, including phospholipid rearrangements, specifically phosphatidylserine relocation to the outer leaflet, and actin-myosin complex contractions [81]. The process begins with ADP-ribosylation factor 6 (ARF6) initiating a sequence of events that leads to the activation of phospholipase D (PLD) [82]. Following this, the extracellular signal-regulated kinase (ERK) is drawn to the plasma membrane, where it phosphorylates and activates the myosin light chain kinase (MLCK) [83]. Subsequently, the activation and phosphorylation of the myosin light chain by MLCK facilitate the release of the MVs [84]. These MVs are characterized by their specific contents, which include ARF6, MHC-I, β1-integrin, VAMP3, and MT1MMP [84]. Other factors inducing MV release include, but are not limited to, calcium influx and hypoxia [85,86,87]. In addition, the ESCRT-I subunit TSG101 has also been reported to be recruited to the plasma membrane by attaching to a tetrapeptide within the Arrestin 1 domain of the protein ARRDC1. This interaction leads to the release of MVs containing TSG101, ARRDC1, and various other cellular proteins [88].

### 3.2. Cargo Composition of EVs

Generally, all EVs contain a mix of different proteins, lipids, and nucleic acids [89,90]. EVs often include proteins key to their genesis, particularly those related to the endosomal pathway, like the ESCRT elements ALIX and TSG101. Proteins crucial for EV genesis and discharge, including RAB27A, RAB11B, and ARF6, are also regularly detected in these vesicles. EVs carry a range of tetraspanins, like CD63, CD81, and CD9, as well as proteins involved in signal transduction (e.g., EGFR), antigen presentation (MHC I and II), and various transmembrane species often in a cell-specific manner (e.g., LAMP1, transferrin receptor) [91,92]. Exosomes are rich in proteins linked to endosome formation, proteins associated with lipid rafts, and heat shock proteins [93,94]. In contrast, MVs are notable for their varied molecular cargo, encompassing a spectrum of proteins, such as integrin receptors, proteases, small GTPases, and multidrug resistance proteins, along with miRNA processing components [95].

Exosomes and MVs exhibit unique lipid compositions that are key to their function and interaction with recipient cells. Research has shown that EVs are generally enriched with sphingomyelin, cholesterol, ganglioside GM3, desaturated lipids, phosphatidylserine, and ceramide [96]. These lipids differ from the typical composition of the plasma membrane of the parental cell, which is usually more rich in phosphatidylcholine and diacylglycerol [97]. Exosomes contain more extracellularly facing phosphatidylserines compared to the cellular plasma membrane, potentially aiding in their uptake and internalization by recipient cells [98,99]. MVs, while similar in lipid composition to their donor cell, are distinctively enriched in polyunsaturated glycerophosphoserine and phosphatidylserine, setting them apart in their lipid profile [90,100]. These distinct lipid compositions are reflective of origin and biogenetic pathways of EVs [89,90].

EVs also contain a wide variety of nucleic acids ranging from mtDNA to small RNAs, with many of them derived from 18S and 28S rRNAs, tRNA, mRNAs, miRNAs, long and short non-coding RNA, piwi-interacting RNA, vault RNA, and Y RNA [101,102,103,104,105,106,107,108,109,110]. These RNA species are selectively incorporated into EVs and have the capacity to be translated in recipient cells, suggesting their role in gene regulation across distant cells [111]. Functional horizontal transfer of EV-loaded endogenous miRNAs via exosomes and MVs has been clearly demonstrated in several scenarios including B-EBV-derived exosomes carrying EBV-miRNA, murine dendritic cell-derived exosomes with miR451, and miR-223 in plasma membrane-derived MVs from monocytes stimulated by granulocyte-macrophage colony-stimulating factor (CSF2) [112,113,114]. In these studies, it was observed that recipient cells, which did not inherently express those miRNAs, showed repressed expression of a target reporter gene affected by the EV-enclosed miRNAs. This underscores the potential of EVs in mediating intercellular communication and gene expression modulation at a distance, rendering them as messengers in horizontal intercellular communication [65].

### 3.3. Characteristics of Stem Cell-Derived EVs

Stem cells are notably prolific producers of EVs, and stem cell-derived EVs (SCEVs) possess a molecular composition that, while sharing the common fundamental structure, diverges from EVs of distinct cell types, influencing their biological functions and therapeutic potentials [115,116]. SCEVs, including those from mesenchymal stromal cells (MSCEVs), are recognized for their rich miRNA composition, which varies depending on the cell type and microenvironment. The active accumulation of miRNA cargo within vesicles can influence recipient cells suggesting dedicated biological roles of these EVs [117,118].

**Figure 1:**
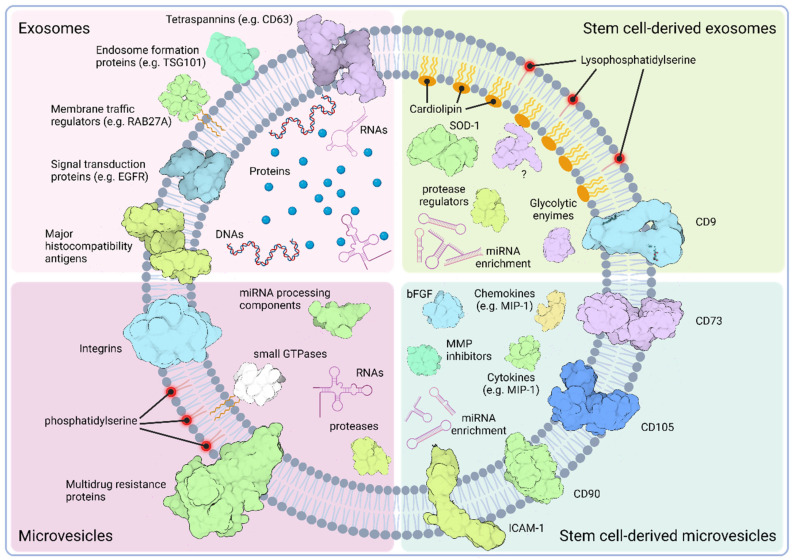
Schematic overview of the characteristic molecular constituents of standard extracellular vesicles and their stem cell-derived counterparts. Exosomes (top left quadrant), generally have their distinctive markers, like tetraspanins (e.g., CD63) and endosome formation proteins (e.g., TSG101 or ALIX), in their membranes. Intravesicularly, they have various protein and nucleic acid cargoes, whereas the former one often includes elements of the endosome genesis and endocytotic pathways. Among nucleic acids, exosomes are often loaded with various RNA components, like the tRNA or rRNA species, and DNA molecules, like the mitochondrial DNA. Standard microvesicles (bottom left quadrant), in contrast, are more enriched with components of the cell membrane and cytoplasm. For the former one, however, alterations compared to the parental cell plasma membrane, like the externalized phosphatidylserine, have been reported. Stem cell-derived exosomes (top right quadrant) show some unique features compared to standard exosomes, like the cardiolipin-enriched inner- and the lysophosphatidylserine-enriched outer leaflet of their membranes. It has also been reported that, among the thousands of cargo proteins identified, they carry enzymes, like the superoxide dismutase-1 (SOD-1) or protease activity regulators (e.g., SERPIN), which are indicative of their regenerative capacity. Their miRNA-enriched cargo together with enzymes influencing the target cells’ glucose metabolism (e.g., aldose reductases) or with uncharacterized functions (e.g., MAGIX) provides an explanation for their multiple effects observed. Stem cell-derived microvesicles (bottom right quadrant) also show some characteristic features compared to standard MVs, including the presence of stem cell-specific surface molecules (e.g., CD105 or CD90), and cargo enriched with growth factors (FGF2), chemokines (e.g., MIP-1), cytokines (e.g., IL-8), extracellular matrix remodeling regulators (e.g., MMP inhibitors), and miRNAs, highlighting the multipurpose physiologic roles of these EVs. Created with BioRender.com.

The lipid bilayer of SCEVs constitutes a mosaic primarily composed of cholesterol, sphingomyelin, glycosphingolipids, and phosphatidylserine, resonating the characteristics of lipid rafts (Figure 1) [119]. Notably, in bone marrow mesenchymal stromal cell-derived extracellular vesicles (BM-MSC-EVs), a discernible lipid dichotomy exists between exosomes and MVs. Exosomes feature an outer layer enriched in lyso-derivatives of phosphatidylserines and free fatty acids, coupled with an inner layer containing cardiolipin. In contrast, MVs emulate the lipid composition of the donor cell’s plasma membrane, accentuating the structural diversity within SCEVs [120].

SCEVs contain specific proteins that reflect their stem cell origin. For instance, SCEVs often express stem cell surface markers, such as CD73, CD90, or CD105 [121,122]. Proteomic studies on SCEVs also revealed EV profiles with wide ranges of cargo proteins [120,122,123,124,125]. Similar to other exosomes, stem cell-derived exosomes also contain proteins related to the MVB biogenesis. However, these may include different members of the tetraspanin family (e.g., CD9 or CD81) that may reflect on the distinct genesis of exosomes depending on the nature of the parental cell. In MSC exosomes, the intravesicular cargo includes enzymes, like superoxide dismutase 1 (SOD-1), which explains why MSC exosomes are regularly reported to alleviate oxidative stress [126]. Stem cell exosomes seem to carry a range of proteins associated with endocytosis, signaling and glycolytic pathways, or peptidase regulation, and even ones with uncharacterized functions (e.g., PDZ domain-containing protein MAGIX) support their wide range of effects upon delivery [127]. MSC-MVs, in contrast, are enriched in cytoskeleton-related elements, like integrins or ICAMs, growth factors (e.g., FGF2), and cyto- and chemokines (e.g., IL-8 and MIP-1, respectively). Data indicate that they are particularly equipped with cargo associated with physiological processes targeting the extracellular matrix, including proteins usually associated with the development of the extracellular matrix (e.g., CD147), tissue remodeling regulators (e.g., CHI3L1) or matrix metalloprotease inhibitors (e.g., TIMP-1) (Figure 1) [122].

SCEVs have emerged as a versatile tool in regenerative medicine, demonstrating therapeutic potential across multiple organ systems [128]. In the nervous system, they are explored for their neuroprotective and neuro-regenerative properties, potentially aiding in recovery from brain trauma and nerve damage [129,130,131]. For respiratory ailments, SCEVs may facilitate lung tissue repair by delivering Angiopoietin-1 mRNA or transferring vascular endothelial growth factor (VEGF), leading to a decrease in pulmonary vascular permeability and proinflammatory cytokine production [132,133]. The broad spectrum of regenerative effects of SCEVs fueled the idea of their potential use in human pathologies affecting tissues with limited endogenous repair capacity, like the myocardium.

## 4. Role of Stem Cell-Derived Extracellular Vesicles in Heart Disease

**Figure 2:**
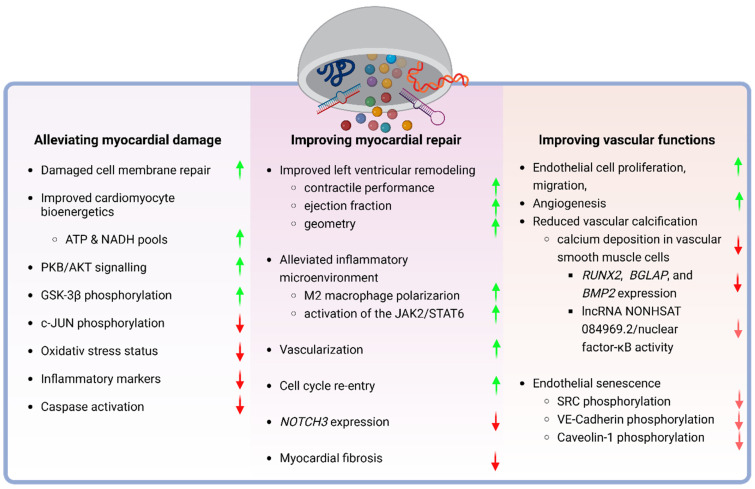
Therapeutic effects of stem cell-derived extracellular vesicles on cardiac and vascular tissue repair. Stem cell-derived extracellular vesicles have therapeutic potential in at least three core areas of cardiovascular diseases. On the one hand, they have been reported to alleviate damage to the myocardium, emphasizing cellular mechanisms, like the repair of damaged cell membranes, enhancement of cardiomyocyte bioenergetics, and regulation of PKB/AKT signaling, with corresponding upregulation (green arrows) or downregulation (red arrows) of biological processes. On the other, they apparently elevate the efficiency of the inherent myocardial repair mechanisms, resulting in improved ventricular remodeling and function, inflammation mitigation, and increased vascularization, alongside the modulation of specific molecular pathways. Data suggest that the beneficial effects of SCEVs on the prevention and repair of myocardial damage are tightly linked to their effects on the vasculature through impacting on endothelial cell functions, angiogenesis, and a reduction in vascular calcification, along with the molecular underpinnings of these effects, highlighting both upregulation and downregulation of critical signaling pathways associated with vascular health and aging. Created with BioRender.com.

Extensive research on stem cells has explored their potential applications in cardiovascular diseases, consistently yielding findings that support the effectiveness of stem cell transplantation upon the diagnosis of heart failure (HF), myocardial infarct (MI), and other cardiac-related conditions (Figure 2). Challenges of stem cell-based therapy in clinical trials, however, prompted exploration of the paracrine effects mediated by SCEVs proving them superior to using stem cells alone in the cardiac context [25].

### 4.1. Dampening the Myocardial Damage

Indeed, exosomes secreted by human embryonic stem cell-derived mesenchymal stromal cells (hESC-MSC-Exo) alleviate key features of ischemia/reperfusion injury (IRI) in the myocardium. In vivo data support the idea that purified hESC-MSC-Exo dramatically reduce infarct size, preserving left ventricular geometry and contractile performance in mice exposed to IRI. Data indicate that these effects are accompanied by changes in the cardiomyocyte bioenergetics, including the increased ATP and NADH levels and elevated phosphorylation of the AKT/PKB and GSK-3β, suggesting a bias toward a pro-survival homeostasis. In concert, an overall decrease in the oxidative stress status with reduced phosphorylation of the stress signaling mediator c-JNK was also observed [134]. These effects are, apparently, not restricted to IRI, since MSC-Exo show beneficial effects in sepsis-induced myocardial injury as well. In this setting, data suggest that effects are, at least in part, mediated by delivering the circular RNA circRTN4 to cardiomyocytes. circRTN4 effectively relieves cardiac injury and apoptosis, suppresses oxidative stress, and reduces inflammatory markers in sepsis-induced rats and lipopolysaccharide (LPS)-treated cardiomyocytes through its regulatory role in the miR-497-5p/MG53 pathway, where circRTN4 acts on miR-497-5p to upregulate MG53 which subsequently repairs damaged cell membranes [135,136]. The role of microRNA as key mediators of the beneficial effects of stem cell-derived EVs is further supported by the findings that cardiac progenitor cell (CPC)-derived exosomes expressing GATA4-responsive-miR-451 effectively protect cardiomyoblasts from ischemia/reperfusion injury-mediated oxidative stress suppressing caspase 3/7 activation. This is in accordance with in vivo data showing reduced cardiomyocyte apoptosis in a mouse IRI model upon CPC-exosome administration, suggesting the therapeutic potential of stem cell-derived exosomes in patients with a high risk of ischemic attack [137].

Experimental findings using EVs from various forms of MSCs and CPCs also suggest that the cardioprotective effects of their exosomes are not limited to the origin of stem cells. This concept is further supported by the observation that exosomes derived from human placental mesenchymal stromal cells (PMSC-Exo) share the cardioprotective trait, showing a comparable capacity to reduce myocardial fibrosis, improve left ventricular remodeling, and reduce both molecular markers of MI and pro-inflammatory indicators in a mouse model [138].

### 4.2. Improvement of the Myocardial Repair

Experimental data also suggest that SCEVs have a therapeutic capacity upon established injury to the myocardium. Indeed, using EVs of Wharton’s Jelly-derived MSCs in a murine myocardial infarction model, significant improvements in the ejection fraction and cardiac output and reduced infarct size were reported [139]. Current experimental data indicate that the myocardial repair capacity of SCEVs is mediated by multiple, possibly parallel, underlying mechanisms. One of the putative mediators is the diverse RNA cargo of SCEVs determined by a number of endo- and exogenous stimuli. The RNA cargo miR-139-3p, for instance, apparently equips SCEVs to modulate the inflammatory microenvironment of the myocardium upon MI. EVs from atorvastatin-pretreated mesenchymal stromal cells (MSCATV-EV) have been reported to be able to shift macrophage polarization toward the M2 state via MSCATV-EV-mediated delivery of miR-139-3p to macrophages and the consequent repression of STAT1 expression [140].

Hypoxic exposure of the parent cells seems to be one of the exogenous factors that enhance the RNA cargo-mediated cardioprotective effects of SCEVs. Indeed, EVs of hypoxic human pluripotent stem cell-derived cardiovascular progenitor cells (PSC-CPC-EVs) show a pronounced capacity for improving cardiac function, reduced fibrosis, enhanced vascularization, and cardiomyocyte survival in the murine myocardium following myocardial infarction [141]. Data indicate that this is, at least in part, mediated by the long noncoding RNA cargo MALAT1 targeting miR-497 for downregulation in recipient cardiomyocytes [141]. A similar mechanism has been observed when using EVs from induced pluripotent stem cell-derived cardiomyocytes after ischemic myocardium insults. These EVs contain distinct sets of cardiomyocyte-specific microRNAs that, upon their delivery, also promote myocardium recovery, resulting in reduced infarct size and pathological hypertrophy [142]. Similar to natural SCEVs, exosomes from induced pluripotent stem cells (iPSC) also show the capacity to facilitate the repair of myocardium, suggesting that this feature is linked to the stem cell nature of the parental cell rather than their origin [25]. Moreover, stimuli, like the hypoxic preconditioning, affect iPCS-derived EVs efficacy in cardioprotection in a similar fashion to that of the ones derived from natural stem cells. Indeed, exosomes from iPSC-cardiomyocytes isolated under hypoxic conditions were also found to significantly improve the viability of the myocardium and enhance cardiac function in a murine myocardial injury model [143]. Hypoxic EVs contain an enriched expression of the miR-106a–363 cluster, which, when transferred, stimulated cardiomyocyte cell cycle re-entry, repressing *NOTCH3* and promoting cell proliferation for myocardial self-repair, suggesting that the hypoxia-triggered conserved adaptative cellular responses can be successfully transmitted to target cells by EV-mediated horizontal communication independently of the origin of EVs [143].

### 4.3. Augmentation of Vascular Functions

Studies on the effects of stem cells in various heart conditions found a consistent association between improved cardiac function and angiogenesis, so one can speculate if the common mechanism behind the beneficial effects of distinct SCEVs on cardioprotection, is, at least in part, the interaction of SCEVs with vascular cells [144,145]. Indeed, SCEVs have been reported to promote angiogenesis and facilitate the proliferation and migration of endothelial cells in the myocardium [146]. This idea is further supported by the observation that EVs released by hypoxic MSCs are taken up readily by endothelial cells leading to enhanced endothelial cell proliferation, migration, and blood vessel formation [147]. Although details of the underlying molecular mechanisms are still to be elucidated, the microRNA cargo of SCEV, as one of the putative mediators, emerged here as well. In both natural aging and type-2 diabetes wound-healing mouse models, MSC-EVs showed beneficial effects via new blood vessel formation. Data indicate that this is mediated by miR-146a, the transfer of which into senescent endothelial cells results in the dephosphorylation of SRC and its downstream targets VE-cadherin and Caveolin-1, ultimately mitigating endothelial cell senescence and stimulating angiogenesis [148]. These data suggest that SCEVs exert their positive effects on endothelial cells by, at least in part, influencing cellular communication among endothelial cells [141]. Since senescent endothelial cells contribute to vascular aging and certain diseases, like atherosclerosis and diabetes, one can also speculate if SCEVs might be exploited for the treatment of vascular pathologies in a wider context.

Indeed, in vitro, BM-MSC-derived exosomes (BM-MSC-Exo) were found to inhibit vascular calcification induced by a high phosphorus concentration in vascular smooth muscle cells (VSMCs). This effect seems to be mediated by the repression of calcification-related genes, like *RUNX2*, *BGLAP*, and *BMP2*, and inhibited calcium deposition in VSMCs via downregulation of the lncRNA NONHSAT 084969.2/nuclear factor-κB axis [149]. Since vascular calcification is one of the pathomechanisms of atherosclerosis, these data suggest the potential use of SCEVs in human pathologies, like hypertension and the consequent cardiac hypertrophy. Indeed, EVs derived from subcutaneous adipose tissue stem cells (ADSC-EVs) or bone marrow-derived mesenchymal stromal cells (BM-MSC-EVs), can significantly reduce the expression of molecular markers associated with cardiac hypertrophy in vitro via their specific microRNA content [150]. Moreover, the former ones have also been reported to be able to reverse hypertension-induced cardiac damage in mice, suggesting a close link between the role of SCEVs in cardiac repair and endothelial homeostasis [151].

To illustrate the intricate nature of the effects of SCEVs in complex cardiovascular pathologies affecting the myocardium and the vasculature alike, systemic administration of MSC-Exo in a doxorubicin-induced dilated cardiomyopathy mouse model has been shown to not only improve cardiac function and reduce cardiomyocyte apoptosis but to decrease the inflammatory cell infiltration as well via modulation of macrophage polarization by the activation of the JAK2/STAT6 pathway [152]. Moreover, it has been observed that human umbilical cord mesenchymal stromal cell-derived EVs (UC-MSC-EVs) reduce autophagy-related protein levels in cardiomyocytes in a type-2 diabetic cardiomyopathy model by inhibition of the AMPK-ULK1 pathway [153]. These data indicate that SCEVs have inherent flexibility to exert comparable beneficial effects in cardiovascular pathologies with a distinct underlying pathomechanism.

## 5. Discussion

Stem cell-derived EVs play pivotal roles in intercellular communication, immune modulation, differentiation, proliferation, and homeostasis regulation [154]. These multifaceted functions have positioned SCEVs at the forefront of regenerative medicine, demonstrating therapeutic benefits across various contexts including cardiovascular diseases [7]. Despite the recent advancements in the understanding and management of cardiovascular diseases, the global burden remains high, with ischemic heart disease as a leading cause of mortality. Moreover, people with certain conditions, like diabetic cardiomyopathy (DCM), a significant contributor to heart failure characterized by complex pathophysiology, still face limited treatment efficacy. This underscores the need for innovative and effective treatment strategies. Stem cell-based cellular therapy, particularly the use of stem cell-derived extracellular vesicles (SCEVs), emerges as a promising avenue in this context.

Indeed, data suggest that the potential of SCEVs in cardioprotection is particularly promising, showcasing their ability to reverse cardiomyocyte damage and promote cardiac repair through diverse mechanisms. Moreover, beyond the strict cardiovascular context, SCEVs could play a role in the treatment of CVD-related secondary pathologies like diabetic nephropathy, via the inhibition of podocyte apoptosis and the promotion of glomerular endothelial cell proliferation [155]. Furthermore, findings that cardioprotective PMSC-Exo also modulate the gut microbiota production of metabolites, like short-chain fatty acids, not only raise the question of a potential link between gut health, inflammation, and the therapeutic effects of PMSC-Exo in MI but also clearly illustrate the suitability of SCEVs in the more systemic approach to CVD management [138].

### 5.1. Challenges of the Use of SCEVs in the Clinical Management of CVDs

Although there has been a great effort to understand the possible place of SCEVs in the clinical management of CVDs, a number of issues delaying the implementation of SCEVs in clinical practice need to be addressed, including scalability and reproducibility. Indeed, one of the major barriers in EV research is the inconsistency in EV characterization across labs due to certain variables, like cell source/type and isolation techniques [156]. This affects the optimization of EV properties, such as concentration, contents, stability, and localization. Although the MISEV initiative by the ISEV has sought to standardize EV definitions and characterizations, standard protocols for both pre-clinical and clinical use of SCEVs in the contexts of CVDs are yet to be developed [46].

Upscaling the production of SCEVs for their use as off-shelf agents in cardiovascular therapies also faces hurdles, since extended passaging of stem cell species for large-scale EV production may result in alterations in their fundamental biological characteristics, including clonal and differentiation abilities [157]. Thus, one can speculate that stem cell senescence may have effects on the cargo composition of SCEV, with fundamental consequences on their beneficial effects in the cardiovascular disease context. This highlights the importance of ex vivo expansion techniques, like the use of hypoxic culture conditions, that have been shown to counteract stem cell senescence, and the careful selection of the initial stem cell population to be used for EV production alike [158].

The latter one is underpinned by observations that the proangiogenic capacity of SCEVs seems to be impaired if they are isolated from patients with cardiovascular risk factors. EVs from adipose tissue-derived mesenchymal stromal/stem cells (AMSCs) of obese patients, for instance, were shown to be less effective than those from lean individuals in mitigating hypertensive cardiomyopathy, raising considerations for the regenerative potential of autologous EVs in certain contexts as well [159].

Indeed, pathologies, like *Diabetes mellitus*, have been shown to have a significant impact on the “EV status” of patients. Erythrocyte-derived EVs are naturally increased in diabetic and pre-diabetic patients in comparison with euglycemic ones, and EVs from patients with type II *Diabetes mellitus* altered the migration and morphology of endothelial cells, contributing to vascular pathologies through increased inflammation [160,161]. Increased inflammation is a risk factor for atherosclerotic peripheral vascular disease in type II *Diabetes mellitus* patients, and this was supported by the observation that these patients have increased EVs containing inflammatory proteins, such as CD5 and higher VEGF-A levels compared to euglycemic controls [161]. The miR-135a-3p cargo of exosomes in type II *Diabetes mellitus* patients has also been proposed to mediate vascular pathologies by activating the ERBB signaling vascular smooth muscle cells, resulting in their promoting abnormal proliferation and migration exacerbating vascular damage [162]. In return, vascular smooth muscle cells release excess amounts of pro-atherosclerotic exosomes that, together with endothelial progenitor cell-derived exosomes, have been found to be enriched in miRNAs, like miR21a-5p, miR-222-3p, and miR-221-5p, that are associated with diabetic atherosclerosis, exacerbating the unfolding of the pathology [163,164]. Findings on the exosome-related paracrine promotion of cardiovascular complications in complex pathologies, like *Diabetes mellitus*, underline the significance of the beneficial effects of SCEVs in CVDs encouraging further exploration of the field [165].

### 5.2. Future Directions

Endothelial EVs can either increase or decrease vascular inflammation based on their protein and noncoding RNA contents [166]. miR-145 and miR-143, for example, maintain vascular smooth muscle cell differentiation, while miR-10a in EVs can inhibit the NF-κB pathway in monocytes, potentially reducing inflammation [167,168]. Conversely, platelet and leukocyte-derived EVs can exacerbate endothelial and monocyte inflammation, promoting thrombosis [169]. Additionally, certain EVs can impair endothelial nitric oxide pathways, reducing vasodilation and contributing to atherosclerosis development [170]. Thus, analysis of cargo composition of circulating EVs may offer insights into CVDs’ presence, severity, and prognosis [171]. EV-derived protein levels of cystatin C, serine protease inhibitors F2, and CD14 have been linked to cardiovascular events, suggesting their potential use as biomarkers in CVDs and, thus, this may contribute to the selection of the most effective SCEVs for therapeutic use [172].

The method of EV delivery is, apparently, another significant barrier to the implementation of EV-based treatment protocols in clinical settings. Indeed, systemic administration of human BM-MSC-derived EVs showed no detectable levels of EVs in the ischemic myocardium compared to intramyocardial delivery in murine ischemia models [173]. Cardiac fibroblasts, which constitute 20% of the heart’s non-myocyte component, might present a solution to the problem of the optimal delivery of therapeutic EVs to the damaged myocardium [174]. Cardiac fibroblasts (CFs), cells typically of mesenchymal origin, are found in myocardial connective tissue and maintain structure and function by producing and secreting extracellular matrix components, such as collagen, elastin, and glycoproteins [175,176,177]. They also play a role in regulating myocardium remodeling during disease by their locally secreted EVs that may carry bioactive molecules, like receptor tyrosine kinases (e.g., DDR2) or proteases (e.g., MMP2), that help regulate various processes, including inflammation, fibrosis, angiogenesis, and cardiomyocyte function [174,178,179,180,181,182]. Cardiac fibroblasts are activated by, and conversely stimulate, pro-inflammatory cytokines, like interleukin-1β (IL-1β) and tumor necrosis factor α (TNFα) to initiate MMP production and migration [183]. Post-injury to the myocardium, CFs with MMP and collagen-containing exosomes migrate to the site of infarction or injury and lead to degradation of the damaged extracellular matrix and re-collagenization of the affected area. Exploiting cardiac fibroblasts as a tissue-resident source of therapeutic EVs faces the challenge that upon myocardium injury, cardiac fibroblasts seem to release endogenous exosomes (CFEV) that are often counterproductive for the heart’s function, leaving behind fibrotic tissue and/or abnormally increasing cardiomyocyte size (hypertrophy) with reduced heart capacity for contraction [180,182]. CFEVs enriched with miRNA passenger strands (“star” miRNA) are typically degraded intracellularly. During and after myocardial injury, however, miR-21*, a star miRNA, acts as a potent mediator of cardiomyocyte hypertrophy [182]. It has also been shown that CFEVs can target angiotensin II receptor type 1 (AT1R) and 2 (AT2R) and chronic activation of the myocardial renin-angiotensin system (RAS), leading to increased levels of angiotensin II (Ang II), which also contributes to cardiac hypertrophy and heart failure [184].

In contrast, CFEVs have been shown to enhance the efficiency of intracellular Ca^2+^ cycling, an important aspect of excitation–contraction coupling, in human iPSC progenitor cardiomyocytes, suggesting an intimate relationship between cardiac stem cell species and the tissue-resident fibroblast population [175]. This raises the question of whether CFEVs can be reprogrammed to be more cardioprotective, an idea that, apparently, is supported by promising results of the use of haemopoietic stem cell- and induced pluripotent stem cell-derived fibroblasts for the repair of the myocardium after myocardial infarction [185,186].

EVs derived from cardiospheres may present another approach for the combination of the advantages of stem cells and tissue-resident fibroblasts. Originating from cardiac surgical biopsy specimens, cardiospheres are spherical, multicellular aggregates formed in culture. These clusters, which emerge from primary tissue cultures, are notable for their high concentration of proliferative cells that exhibit stem cell markers, as well as other cells that spontaneously differentiate into cardiac lineages [187]. From these cell clusters, cardiac progenitor cells (CDC) can be isolated, and their EVs markedly enhance cardiac function post-MI [188]. Data indicate that CDC-EVs also have the ability to improve the angiogenic capacity of M1 macrophages, leading to significant increases in tube formation and branching in in vitro angiogenesis assays [189]. Further characterization of the CDC-derived EVs composition would reveal to what degree they share cargo with other SCEVs which possess similar therapeutic effects. This will bring us closer to the era of a new class of off-shelf, SCEV-based therapeutics used in the clinical managements of cardiovascular diseases.

## 6. Conclusions

Current data indicate that stem cell-derived extracellular vesicles present a very promising new direction for the more successful treatment of cardiovascular diseases. However, further research on the mechanism of EV uptake by cardiac cells and the interplay between cardiac myocytes and their microenvironment is critical for exploring avenues towards personalized EV therapies based on individual profiles and the advancement of the SCEV-based regenerative medicine in cardiology.

## 7. Glossary

ARF6—ADP-ribosylation factor 6 is a small GTPase protein that plays a role in regulating the movement of vesicles and various cellular processes, such as intracellular vesicular trafficking and membrane dynamics.

ATP—A compound that transports energy within cells, acting as the principal energy currency for diverse cellular functions. It liberates energy upon the hydrolysis of its phosphate bonds during cellular activities, including muscle contraction and biochemical reactions.

AMPK-ULK1—A signaling cascade in the regulation of autophagy, which is a cellular process that degrades and recycles damaged or unnecessary cellular components. The pathway is important for maintaining cellular homeostasis and removing dysfunctional cellular components.

Cardiac remodeling—The structural and functional changes that occur in the heart in response to various stimuli, often as a result of chronic stress, injury, or disease. These changes can affect the size, shape, and function of the heart, and they may be adaptive (i.e., during pregnancy or regular exercise) or maladaptive (pathological conditions, like MI) depending on the context.

Cardiomyocytes—Specialized muscle cells that form the muscular tissue of the heart, known as the myocardium. These cells are responsible for the contraction and pumping action of the heart, allowing it to circulate blood throughout the body. Cardiomyocytes are a vital component of the heart and play a central role in maintaining cardiovascular function. Their capacity for self-renewal is limited.

Dilated cardiomyopathy—A heart muscle disorder characterized by the enlargement of the heart chambers, particularly the left ventricle. As the heart chambers become enlarged, the muscle wall weakens, leading to impaired pumping of blood and an inability for the heart to contract forcefully. This condition can result in heart failure, where the heart is unable to effectively meet the body’s demands for oxygenated blood.

Ejection fraction—A measure of the percentage of blood that is pumped out of the heart’s left ventricle with each contraction and into the rest of the body. It is a widely used parameter in assessing cardiac function and is often used to evaluate heart health. The ejection fraction provides information about how effectively the heart is pumping and how well it can maintain adequate blood circulation. A normal ejection fraction is typically between 50% and 70%. A lower ejection fraction may indicate weakened heart function and can be associated with conditions, such as heart failure, cardiomyopathy, or other cardiac issues.

Embryonic stem cells—These cells may be categorized as toti- or pluripotent based on their ability to generate cells of the placenta and umbilical cord. Toti- and pluripotent ESCs represent the pinnacle of differentiation, holding immense potential within stem cell therapy. Unlike somatic stem cells, which undergo cellular senescence, ESCs are considered immortal, partially attributed to their elevated telomerase expression.

ESCRT—The endosomal sorting complex for transport is a group of protein complexes that play a crucial role in various cellular processes, particularly in the endocytic pathway. The endocytic pathway involves the internalization of substances into a cell through the formation of vesicles derived from the cell membrane.

Extracellular matrix—An intricate network comprising proteins and carbohydrates that furnishes structural and biochemical support to adjacent cells. The extracellular matrix resides in the intercellular spaces within tissues and organs, actively shaping their overall structure and function. Significantly, the ECM participates in cell signaling, adhesion, and fundamental cellular activities like migration and differentiation. Additionally, it plays a role in the development, maintenance, and reparative processes of tissues.

Heart failure—A medical condition in which the heart is unable to pump blood effectively, leading to inadequate circulation of blood and insufficient delivery of oxygen and nutrients to the body’s tissues. Despite its name, heart failure does not mean that the heart has completely stopped working; rather, it indicates that the heart’s pumping ability is compromised. Symptoms commonly include fatigue, shortness of breath, and edema.

IRI—Ischemia/reperfusion injury occurs when blood flow to a tissue or organ is temporarily restricted (ischemia) and then restored (reperfusion). This process can result in additional damage to the tissue during the reperfusion phase, exacerbating the injury caused by the initial ischemia. Ischemia/reperfusion injury is a common occurrence in various clinical situations, including MI, stroke, and certain surgical procedures.

LDL cholesterol—Often referred to as “bad cholesterol”, it is a type of lipoprotein that transports cholesterol from the liver to the cells in the body. While cholesterol is essential for various bodily functions, including the production of hormones and cell membranes, too much LDL cholesterol in the blood can contribute to the development of atherosclerosis.

Lipid raft—A specialized area within the cell membrane that is rich in cholesterol, sphingolipids, and proteins. Lipid rafts are dynamic and heterogeneous structures that play a role in various cellular processes, including signal transduction, membrane trafficking, and cellular adhesion. They are also important in immune cells, where they contribute to the organization of signaling molecules.

MHC—A cluster of genes responsible for encoding proteins essential to immune system functionality and the presentation of antigens to T cells, facilitating immune recognition. MHC class I and class II molecules fulfil specific roles in presenting intracellular and extracellular antigens, respectively, thereby influencing immune responses and compatibility in transplantation.

MT1MMP—Membrane-type 1 matrix metalloproteinase is a membrane-bound enzyme that belongs to a group of proteolytic enzymes involved in the breakdown of the extracellular matrix. MT1-MMP plays a crucial role in ECM remodeling and is associated with various physiological and pathological processes, such as angiogenesis and cancer formation, respectively.

Myocardial infarct—The obstruction of blood flow to a segment of the cardiac musculature, typically induced by a thrombus, resulting in injury or necrosis of the myocardial tissue. Termed colloquially as a “heart attack,” manifestations encompass chest pain, dyspnea, and discomfort in the upper thoracic region.

Senescence—A condition where cells cease dividing irreversibly and undergo functional changes, commonly linked to aging or cellular stress. Additionally, senescent cells have the potential to release factors that promote inflammation and affect nearby tissues.

STEMI—A type of heart attack distinguished by a prolonged blockage of blood flow to the cardiac muscle, discernible through distinctive alterations in the ECG, namely ST-segment elevation. STEMI is recognized as a medical emergency, emphasizing the imperative need for swift intervention to reinstate blood circulation and mitigate harm to the myocardium.

Paracrine effects—The local signaling effects that occur between neighboring cells. In paracrine signaling, cells release signaling molecules (such as growth factors, cytokines, or neurotransmitters) into the extracellular fluid, and these molecules act on nearby target cells. Unlike endocrine signaling, which involves the release of signaling molecules (hormones) into the bloodstream for distant target cells, paracrine signaling is more localized.

VEGF—A signaling protein that contributes to the development of new blood vessels and the upkeep of existing ones. It plays a crucial role in diverse physiological processes, such as embryonic development, wound healing, and the female reproductive system. Moreover, VEGF is associated with pathological conditions, such as tumor angiogenesis.

Ventricular geometry—A term that refers to the shape and structure of the ventricles, the two lower chambers of the heart responsible for pumping blood to the lungs and the rest of the body. The ventricular geometry is an important aspect of cardiac anatomy and function, influencing the efficiency of blood flow and the heart’s ability to pump blood effectively. Ventricular geometry is linked to cardiac output and stroke volume, and can be visualized through echocardiography, magnetic resonance imaging, and computed tomography scans to assess function.

## Supplementary Materials

The following supporting information can be downloaded at: https://www.mdpi.com/article/10.3390/pharmaceutics16030381/s1, File S1: Isolation of extracellular vesicles.

## List of Abbreviations

ADSC-EVs	Adipose tissue stem cell-derived extracellular vesicles
AMSCs	Adipose tissue-derived mesenchymal stromal/stem cells
ANGII	Angiotensin II
ARF6	ADP-ribosylation factor 6
AT1R	Angiotensin receptor 1
AT2R	Angiotensin receptor 2
BM-MSCs	Bone marrow-derived mesenchymal stromal/stem cells
BM-MSCEVs	Bone marrow mesenchymal stromal cell-derived extracellular vesicles
BM-MSC-Exo	Bone marrow mesenchymal stromal cell-derived exosomes
BM-SCEVs	Bone marrow-derived stem cell extracellular vesicles
CA	Cardiac arrest
CABG	Coronary artery bypass graft
CF	Cardiac fibroblasts
CD	Cluster of differentiation (a biomarker)
CFEV	Cardiac fibroblast-released extracellular vesicles
CHD	Coronary heart disease
CPC	Cardiac progenitor cell
CVD	Cardiovascular disease
DCM	Dilated cardiomyopathy
DDR2	Discoidin domain receptor 2
DMCM	Diabetic/Diabetes Mellitus cardiomyopathy
DM-HCAEC	Diabetes Mellitus human coronary artery endothelial cells
ERBB	Avian erythroblastic leukemia viral oncogene homolog receptor tyrosine kinase
ERK	Extracellular signal-regulated kinase
ESCRT	Endosomal sorting complex required for transport
EV	Extracellular vesicles
FBS	Fetal bovine serum
G-CSF	Granulocyte-colony stimulating factor
hBM-MSCEVs	Human bone marrow mesenchymal stromal cell-derived extracellular vesicles
hESC-MSC-Exo	Human embryonic stem cell mesenchymal stromal cells derived exosomes
HF	Heart failure
hPMSC-Exo	Human placental mesenchymal stromal cell exosomes
HSCs	Hematopoietic stem cells
hUCMSC-Exo	Human umbilical cord mesenchymal stromal cell exosomes
ILVs	Intraluminal vesicles
ISEV	International Society of Extracellular Vesicles
IRI	Ischemia/reperfusion injury
iPSCs	Induced pluripotent stem cells
iPSC-CMs	Induced pluripotent stem cell-derived cardiomyocytes
iPSC-EVs	Induced pluripotent stem cell-derived extracellular vesicles
JAK1	Janus kinase 1
LPS	Lipopolysaccharide
MI	Myocardial infarct
miRNA	Microribonucleic acid
MISEV	Minimal information for studies of extracellular vesicles
MLCK	Myosin light chain kinase
MMP2	Matrix metalloproteinase 2
MSCs	Mesenchymal stromal cells
MSCATV-EV	Atorvastatin-pretreated mesenchymal stromal cell extracellular vesicles
MSCEVs	Mesenchymal stromal cell-derived extracellular vesicles
MSC-Exo	Mesenchymal stromal cell-derived exosomes
MVBs	Multivesicular bodies
MVs	Microvesicles
NSCs	Neural stem cells
NVs	Nanovesicles
PDE-5	Phosphodiesterase-5
PLD	Phospholipase D
PLT	Human platelet lysate
PSC-EVs	Human pluripotent stem cell-derived cardiovascular progenitor extracellular vesicles
RAS	Renin angiotensin system
SCEVs	Stem cell-derived extracellular vesicles
STEMI	ST-segment elevation myocardial infarction
TGF-β1	Transforming growth factor-β1
VEGF	Vascular endothelial growth factor
VSMCs	Vascular smooth muscle cells
