# Supplementary material for: Stem Cell-Derived Extracellular Vesicles in the Treatment of Cardiovascular Diseases
Source: Pharmaceutics. 2024 Mar 11;16(3):381. doi: 10.3390/pharmaceutics16030381 (PMC10974254; doi:10.3390/pharmaceutics16030381)
Supplement: Supplementary file 1 [file pharmaceutics-16-00381-s001.zip › pharmaceutics-2872705-supplementary.pdf]

Supplementary information

Isolation of extracellular vesicles

The following generic protocol for purification of extracellular vesicles has been originally described by Théry et al. [47].

Step 1. Primary centrifugation

Collect cells by centrifugation in medium relevant to the cells of interest at 300 g for 10 minutes at 4°C. Collect supernatant and subject it to step 2.

Step 2. Secondary centrifugation

Centrifuge supernatant from step 1 at 2,000 g for 10 minutes at 4°C. Collect supernatant and subject it to step 3.

Step 3. Intermediate centrifugation

Centrifuge supernatant from step 2 at 10,000 g for 30 minutes at 4°C. For purification of small extracellular vesicles, collect supernatant and subject it to step 4. For isolation of microvesicles collect the pellet from this step.

Step 4. Primary ultracentrifugation

Centrifuge supernatant from step 3 at 100,000 g for 70 minutes at 4°C using an ultracentrifuge, collecting small EVs, including exosomes, in the pellet.

Step 5. Pellet Resuspension

Discard supernatant from step 4 and resuspend in 1x PBS or 1x TBS the pellet that contains EVs along with contaminating proteins. Proceed to step 6.

Step 6. Secondary ultracentrifugation

Perform ultracentrifugation of the resuspended fraction from step 5 at 100,000 g for 70 minutes at 4°C to wash the small EVs/exosomes. Discard supernatant and resuspend the pellet in 1x PBS or 1x TBS.
